# *Notes from the Field*: Characteristics and Monitoring of the 2026 Outbreak of Ebola Disease Caused by Bundibugyo Virus — Democratic Republic of the Congo, August 2026

**DOI:** 10.15585/mmwr.mm7535e1

**Published:** 2026-09-10

**Authors:** Dumazedier Kabasele, Erika Meyer, Issaka Kabore, Amber Dismer, Joelle K. Kabamba, Anna Bratcher, Constantin Kabwe Kola, Carrie Eggers, Delayo Zomahoun, Noemi Hall, Mutshiene Deogratias Ekwanzala, Natalie Peters, Amy Schuh, Tara Sealy, Philip Ricks, Billy Mpianga Mutombo, Michael Kinzer, Benjamin A. Dahl, Hyacinte Kabore, Mary J. Choi, John Rossow, Sascha Ellington, CDC 2026 Ebola Response International Epidemiology and Laboratory Task Force

**Affiliations:** ^1^CDC 2026 Ebola Response; ^2^U.S. Public Health Service, Rockville, Maryland.

**Summary**

**What is already known about this topic?**

In May 2026, an outbreak of Ebola disease caused by Bundibugyo virus was identified in the Democratic Republic of the Congo.

**What is added by this report?**

This ongoing outbreak is now the second largest Ebola outbreak in history. The targets for five critical public health response indicators (case detection alerts, contact tracing, laboratory testing, isolation of infected persons, and safe and dignified burials) have not yet been met, and the outbreak continues to expand rapidly.

**What are the implications for public health practice?**

Substantial improvements in established outbreak control measures are crucial to rapidly detect and diagnose cases and isolate and provide treatment for infected persons, prevent funeral-associated transmission to prevent additional spread, and control this rapidly expanding outbreak.

The Democratic Republic of the Congo (DRC) Ministry of Public Health declared an Ebola outbreak on May 15, 2026 (*1*). Two days later, CDC activated its Emergency Operations Center as part of the U.S. government response to this rapidly growing outbreak (*2*). This report describes the epidemiologic characteristics and monitoring of the ongoing outbreak in DRC.

## Investigation and Outcomes

### Background

The 2026 Ebola DRC outbreak caused by Bundibugyo virus is now the second largest Ebola outbreak ever recorded. As of August 21, 2026, DRC reported 5,458 confirmed cases and 2,606 (48%) confirmed deaths. Compared with previous Ebola outbreaks, the increase in cases in DRC is unprecedented, with approximately 5,000 cases in 100 days (Ebola Outbreak: Current Situation | CDC). Cases have been reported from six of the 26 DRC provinces (Bas-Uélé, Haut-Uélé, Ituri, North Kivu, South Kivu, and Tshopo), affecting 57 of 151 health zones in the affected provinces. Ituri province remains the outbreak epicenter, accounting for 84% of reported cases. Strategies known to control Ebola outbreaks include community-based surveillance, case detection alert notifications,* rapid and in-depth case investigations, identification and monitoring of contacts, infection control measures (e.g., prompt isolation of persons with suspected or confirmed Bundibugyo virus disease [BVD]), rapid diagnostic testing, mortality surveillance, and safe and dignified burials (SDBs).^†^

### Data Source

Operational indicators for five domains have been generated based on experience with previous Ebola outbreaks, including DRC’s 2018 outbreak (*3*) (Table). Targets reflect the levels necessary to end the outbreak. The DRC Ministry of Public Health prepares publicly available daily situation reports, and CDC abstracts data from these reports to evaluate the established indicators each day. Indicator data are monitored over time to assess the outbreak trajectory. This activity was reviewed by CDC, deemed not research, and conducted consistent with applicable federal law and CDC policy.^§^

**TABLE:** Public health objectives, operational indicators, and status of reaching targets to end the Ebola outbreak caused by Bundibugyo virus — Democratic Republic of the Congo, August 2026

Domain	Public health objective	Indicator*	Target	Status as of August 21, 2026
Case detection alerts^†^	Detect cases rapidly	Percentage of alerts investigated within 24 hours (3-wk average)	>90% of alerts investigated	Not available^§^
Contact tracing	Interrupt disease transmission	Average no. of contacts identified per case (3-wk average)	≥20 contacts identified per case	10.6 contacts identified per case^¶^
		Daily percentage of contact tracing completeness (3-wk average)	>95% complete	82% complete^¶^
		Percentage of new cases that are known contacts (3-wk average)	>90% of new cases	No data
Laboratory testing	Identify persons with laboratory-confirmed Ebola disease	Percentage of validated alerts with laboratory testing for Ebola (3-wk average)	>90% tested	72% tested^¶^
		Percentage of laboratory tests for Ebola that are positive (3-wk average)	0% positive	24% positive^¶^
Isolation	Prevent spread of infection	Percentage of ETU beds occupied (3-wk average)	<80% occupied both for isolation beds and for treatment beds	64% occupied^¶^
		Percentage of persons with confirmed BVD isolated within 24 hours (3-wk average)	>90% isolated	Not available^¶,^**
SDB	Prevent funeral-associated transmission	Percentage of confirmed BVD deaths that occur outside an ETU (3-wk average)	0% of deaths	59% of deaths^¶^
		Percentage of affected health zones with an SDB team (cumulative estimate)^††^	100% of health zones	49% of health zones
		Percentage of deaths that receive SDBs	100% of deaths	No data

**Abbreviations**: BVD = Bundibugyo virus disease; ETU = Ebola treatment unit; SDB = safe and dignified burial.

* Data reported or derived from publicly available situation reports from the Democratic Republic of the Congo.

^†^ A case detection alert notification is generated for any of the following: 1) any person with onset of fever and no response to treatment for usual causes of fever in the area; 2) any person with at least one of the following signs: bleeding, bloody diarrhea, or blood in urine; or 3) any sudden death (Ebola Virus Outbreak Toolbox | World Health Organization).

^§^ Last reported on August 5, 2026, as 83% (3-week average); indicated as not available because a more recent estimate is not available.

^¶^ The 3-week period for calculating these indicator values was July 31–August 21, 2026.

** Last reported on July 12, 2026, as 15%–20% overall; indicated as not available because a more recent estimate is not available.

^††^ Reported from implementing partners FHI 360 on August 19, 2026, and the International Federation of Red Cross and Red Crescent Societies on August 20, 2026.

### Operational Indicator Analysis

Nearly all operational indicators remain below identified targets (Table). Operational indicator values were calculated for the 21-day period of July 31–August 21. The average percentage of alerts investigated within 24 hours (last reported August 5, 2026) was 83% (target = >90%). An average of 10.6 contacts were identified per confirmed case (target = ≥20), suggesting underreporting and underascertainment of case contacts. The percentage of confirmed new cases previously identified as known contacts (last reported July 12, 2026) was 15%–20% (target = >90%); this suggests that most cases are occurring outside known transmission chains. In addition, more than one half (59%) of confirmed Ebola deaths are occurring outside an Ebola treatment unit (ETU) (target = 0%), suggesting insufficient ETU capacity, fear of ETUs, and ongoing spread through unidentified transmission chains. Laboratory testing was performed for 72% of validated alerts (target = >90%), indicating that a substantial number of suspected cases remain untested. Test positivity was 24%, with a target of 0%. Although the national ETU bed occupancy was 64%, meeting the target of <80%, occupancy varied substantially by health zone, with some facilities unable to isolate all infected persons and reporting occupancies as high as 140%. Fewer than one half (49%) of affected health zones had at least one SDB team (target = 100%). Current data were not available for several response indicators, such as percentage of persons with confirmed BVD receiving prompt isolation (target = >90%) and percentage of deaths with SDBs (target = 100%), underscoring ongoing data gaps in this complex public health response.

## Preliminary Conclusions and Actions

As of August 21, 2026, most operational indicator measures remained below established response targets, and data for others were unavailable, indicating gaps in surveillance, contact tracing, laboratory testing, health care–seeking, isolation, and SDB capacity that limit control of the ongoing outbreak. These missing data and operational gaps, together with continued geographic expansion of the outbreak, a high percentage of deaths occurring outside ETUs, and a low percentage of cases among persons previously identified as contacts, indicate uncontrolled expansion of the outbreak. Public health response activities are complicated by a protracted complex humanitarian emergency in the eastern part of DRC, including armed conflict, limited health infrastructure, population displacement and mobility, and constraints on access to affected communities.

Containment and control of the 2026 Ebola disease outbreak requires integration and coordination of at least five response areas: 1) expansion of community-based surveillance systems ensuring rapid investigation of alerts; 2) improvements in contact tracing completeness and timeliness; 3) expansion of treatment and isolation capacity in affected health zones; 4) increased laboratory testing capacity, enabling prompt case identification; and 5) ensuring SDBs in affected health zones.

In addition, collecting robust, high-quality data regarding these operational actions is essential at the health zone level; CDC’s continued support to the DRC Ministry of Public Health and partners with improving data collection is critical. Collecting data at the level of the health zone facilitates timely local outbreak response decisions. Rapidly enhancing international humanitarian coordination and mobilizing global technical, operational, and other needed support are critical for accelerating the response and controlling the outbreak.

## Footnotes

* A case detection alert notification is generated for any of the following: 1) any person with onset of fever and no response to treatment for usual causes of fever in the area; 2) any person with at least one of the following signs: bleeding, bloody diarrhea, or blood in urine; or 3) any sudden death.

† An SDB is the safe management, preparation, transport, and burial of the body of a person who has died from suspected or confirmed Ebola disease. Trained personnel conduct the burial using appropriate infection prevention and control measures to prevent exposure and transmission while also respecting the dignity of the decedent and accommodating the cultural, religious, and personal wishes of the family and community to the extent that they can be carried out safely.

§ 45 C.F.R. part 46.102(l)(2), 21 C.F.R. part 56; 42 U.S.C. Sect. 241(d); 5 U.S.C. Sect. 552a; 44 U.S.C. Sect. 3501 et seq.
